# Molecular Subtypes and Precision Oncology in Intrahepatic Cholangiocarcinoma

**DOI:** 10.3390/jcm10132803

**Published:** 2021-06-25

**Authors:** Carolin Czauderna, Martha M. Kirstein, Hauke C. Tews, Arndt Vogel, Jens U. Marquardt

**Affiliations:** 1 Department of Medicine I, University Medical Center Schleswig-Holstein—Campus Lübeck, 23562 Lübeck, Germany; carolin.czauderna@uni-luebeck.de (C.C.); Martha.Kirstein@uksh.de (M.M.K.); hauke.tews@uksh.de (H.C.T.); 2 Departement of Internal Medicine I, University Hospital Regensburg, 93042 Regensburg, Germany; 3 Department of Gastroenterology, Hepatology and Endocrinology, Hannover Medical School, 30625 Hannover, Germany; Vogel.Arndt@mh-hannover.de

**Keywords:** cholangiocarcinoma, FGFR, IDH1, targeted therapy, immunotherapy

## Abstract

Cholangiocarcinomas (CCAs) are the second-most common primary liver cancers. CCAs represent a group of highly heterogeneous tumors classified based on anatomical localization into intra- (iCCA) and extrahepatic CCA (eCCA). In contrast to eCCA, the incidence of iCCA is increasing worldwide. Curative treatment strategies for all CCAs involve oncological resection followed by adjuvant chemotherapy in early stages, whereas chemotherapy is administered at advanced stages of disease. Due to late diagnosis, high recurrence rates, and limited treatment options, the prognosis of patients remains poor. Comprehensive molecular characterization has further revealed considerable heterogeneity and distinct prognostic and therapeutic traits for iCCA and eCCA, indicating that specific treatment modalities are required for different subclasses. Several druggable alterations and oncogenic drivers such as fibroblast growth factor receptor 2 gene fusions and hotspot mutations in isocitrate dehydrogenase 1 and 2 mutations have been identified. Specific inhibitors have demonstrated striking antitumor activity in affected subgroups of patients in phase II and III clinical trials. Thus, improved understanding of the molecular complexity has paved the way for precision oncological approaches. Here, we outline current advances in targeted treatments and immunotherapeutic approaches. In addition, we delineate future perspectives for different molecular subclasses that will improve the clinical care of iCCA patients.

## 1. Introduction

**Figure 1:**
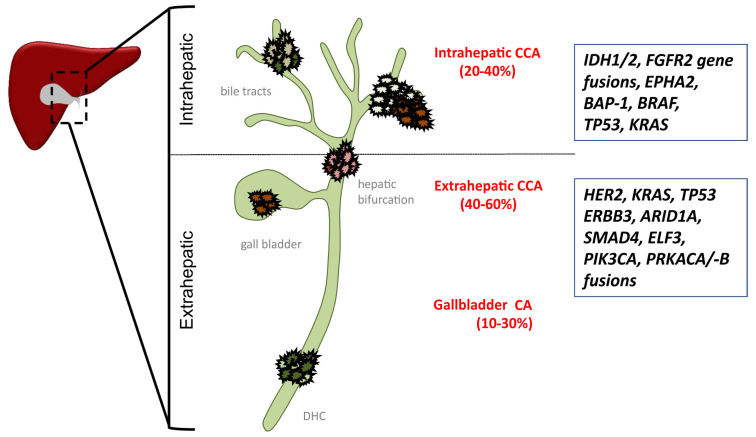
Molecular alterations of cholangiocarcinoma (CCA) subtypes. AT-Rich Interaction Domain 1A (AIRD1A); BRCA1 Associated Protein 1 (BAP-1); proto-oncogene B-Raf (BRAF); Ductus hepaticus communis (DHC); human epidermal growth factor receptor 2 (Her2); E74 Like ETS Transcription Factor 3 (ELF3); Ephrin type-A receptor 2 (EPHA2); Erb-B2 Receptor Tyrosine Kinase 3 (ERBB3); Fibroblast Growth Factor Receptor gene 2 (FGFR 2), Isocitrate dehydrogenase 1 and 2 (IDH1/2); Kirsten rat sarcoma (KRAS); Phosphatidylinositol-4,5-Bisphosphate 3-Kinase Catalytic Subunit Alpha (PIK3CA); Protein Kinase CAMP-Activated Catalytic Subunit Alpha/-Beta (PRKACA/-B); SMAD family member 4 (SMAD4); Tumor Protein P53 (TP53).

Biliary tract cancer (BTC) represents the second-most common primary liver malignancy, with an estimated incidence rate of 6/100,000. BTC can be classified by anatomic localization into intrahepatic cholangiocarcinoma (iCCA), perihilar and distal or extrahepatic cholangiocarcinoma (eCCA), and gallbladder carcinoma (GBCA) (Figure 1). Surgical resection is the only potentially curative treatment approach. However, most patients are diagnosed at advanced stages, when surgical and loco-regional therapies are not feasible, which overall renders BTC prognostically dismal [1,2]. Even following R0-resection, recurrence rates are high, with 3-year recurrence-free survival (RFS) rates below 30% [3]. At advanced stages, median overall survival (mOS) is historically described not to exceed 12 months in unselected patients, underlining the urgent need for effective systemic therapies [4].

Several systemic treatments have been evaluated to improve the RFS and OS for patients with CCA in different clinical scenarios. Gemcitabine-based adjuvant therapies have been tested vs. observation, the former standard of care, within the phase III BCAT and Prodige-12-Accord-18 trials in the context of adjuvant therapeutic settings [5,6]. Both studies failed to meet their primary endpoint, implying that gemcitabine should not be given in the adjuvant setting outside of clinical trials. The phase III BILCAP trial pursued an adjuvant approach with the oral fluoropyrimidine (FU) capecitabine in patients with BTC, following resection [7]. The primary endpoint mOS was numerically but not statistically prolonged in the intention-to-treat (ITT) population (51.1 vs. 36.4 months; HR = 0.81, 95% CI = 0.63–1.04; *p* = 0.097). However, in the per-protocol population, improvement reached statistical significance (52.7 vs. 36.1 months; HR = 0.75; 95% CI = 0.58–0.97; *p* = 0.028). Additionally, the secondary endpoint mRFS was improved in the ITT population (24.4 vs. 17·5 months; HR = 0.76; 95% CI = 0.58–0.99; *p* = 0.039). Despite the formally negative result of the BILCAP trial, a survival benefit can be anticipated by adjuvant treatment with capecitabine and has, therefore, become the standard of care in many centers. Accordingly, the current American Society of Clinical Oncology (ASCO) guidelines implemented a statement to propose for patients with resected CCA adjuvant chemotherapy with capecitabine [8].

In the palliative setting, chemotherapy with gemcitabine and cisplatin represents the standard in the first-line treatment for unselected patients with recurrence after resection or with primarily advanced-stage disease since 2010 based on the results of the phase III ABC-02 trial [4]. In this study, the mOS was improved from 8.1 to 11.7 months with gemcitabine and cisplatin compared to gemcitabine alone (HR = 0.64; 95% CI = 0.52–0.80; *p* < 0.001). A more intensive chemotherapy with modified FOLFIRINOX was recently tested in first-line therapy against the standard gemcitabine and cisplatin within the phase II Amebica-Prodige-38 trial [9]. Disappointingly, the primary endpoint was not reached in the FOLFIRINOX arm, with a 6-month progression-free survival (PFS) rate of 44.6% compared to 59.0% in the gemcitabine and cisplatin arm. In contrast, the phase II single-arm NCT02392637 trial achieved a promising median PFS (mPFS) of 11.8 months with triplet chemotherapy with gemcitabine, cisplatin, and nab-paclitaxel [10]. These findings will be further tested within a phase III trial against gemcitabine and cisplatin (ClinicalTrials.gov Identifier: NCT03768414).

In second-line settings, there was a lack of phase III data for a long time. In clinical practice, however, most often, FU-based chemotherapy has been used following failure to treatment with gemcitabine and cisplatin. Recently, the phase III ABC-06 trial investigated chemotherapy with FOLFOX vs. active symptom control (ASC) [11]. The primary endpoint mOS was significantly prolonged in the FOLFOX arm (6.2 vs. 5.3 months; HR = 0.69; 95% CI = 0.50–0.97; *p* = 0.031). Patients who progressed rapidly during first-line therapy with gemcitabine and cisplatin did not benefit from subsequent therapy with FOLFOX. Despite the formally positive result, the clinical relevance remains questionable regarding a survival benefit of less than a month against BSC and certainly warrants the need for more effective treatment strategies in second-line therapy.

Despite positive results of both phase III ABC-02 and ABC-06 trials for unselected patients with BTC, the survival benefits of chemotherapy remain modest and highlight the necessity of improving systemic treatments. Several lessons can be learned from the different trials. In particular, it seems unlikely that with the currently available chemotherapy options, survival will be clinically meaningfully improved in all-comer settings, without selection of patients according to their localization as well as molecular profile. Importantly, our increasing understanding of the molecular alterations that drive cholangiocarcinogenesis highlights the genomic heterogeneity of CCAs and, thus, provides a clear rationale for molecular targeted and personalized treatment approaches [12].

Comprehensive molecular characterization with translation to biomarker-based clinical trials mainly exists for iCCA, while molecular data of eCCA are still limited. The current review will, therefore, outline recent advances in molecular characterization and subgrouping of iCCA, with a focus on translational and novel therapeutic approaches that explore the sizeable number of oncogenic alterations in this deadly disease.

## 2. Molecular Basis and Emergence of Druggable Alterations of iCCA

Over the recent years, it has become increasingly clear that tumor development of iCCA, similar to hepatocellular carcinoma (HCC), is frequently caused by underlying chronic inflammatory damage and changes in the tissue microenvironment [13,14,15]. The resulting profound phenotypic and molecular heterogeneity, as well as the diverse etiological types of underlying chronic liver inflammation, induce an adverse oncogenic niche capable of promoting malignant transformation. Herein, identified molecular profiles of different iCCA subtypes resemble activation of distinct signaling pathways involved in inflammation and proliferation [16]. iCCA with dominant activation of pro-inflammatory networks is characterized by induction of immune-related signaling pathways. In contrast, tumors of the proliferation subclass harbor enrichment in classic oncogenic pathways, including deregulated receptor tyrosine kinase (RTK) signaling, RAS–RAF–ERK, PI3K–AKT–mTOR, insulin growth factor receptor 1, *KRAS* mutations, as well as activation of stemness traits. In agreement with a potential translational implication, prognosis of these two subclasses is considerably different. Herein, prognosis of iCCA with proliferative characteristics is a significantly poorer survival than inflammatory cancers. Furthermore, activation of distinct oncogenic driver genes predominantly in the proliferation subtypes vs. immune response signatures within the inflammation subclass highlights the therapeutic implications of molecular profiling.

Several studies suggest that up to 40% of patients with iCCA present with targetable molecular alterations. The most frequent potentially targetable alterations in iCCA include IDH1 (20%), TP53 (20%), and FGFR2 gene fusions (14%), whereas alteration for BRAF (5%), HER2 (8%), and PIK3CA (7.0%) are more frequently found in eCCA [17,18,19,20,21] (Figure 1). Thus, FGFR2 fusions and alterations of IDH1 are the most promising molecular targets in iCCA.

As one of the first biomarker-driven trials, the prospective Mosacto-01 trial provided evidence that molecular targeted treatments can improve survival [17]. Among 1035 patients with advanced solid tumors, 43 patients with BTC were included. Potentially druggable targets could be identified in 68% of the BTC patients. Interestingly, patients who received matched molecular targeted agents had both a prolonged PFS (1.3-fold) and a prolonged mOS (17 vs. 5 months; HR = 0.29; 95% CI = 0.11–0.76; *p* = 0.008) compared to patients who were not treated with a matched targeted therapy according to the molecular profile of their tumors.

**Figure 2:**
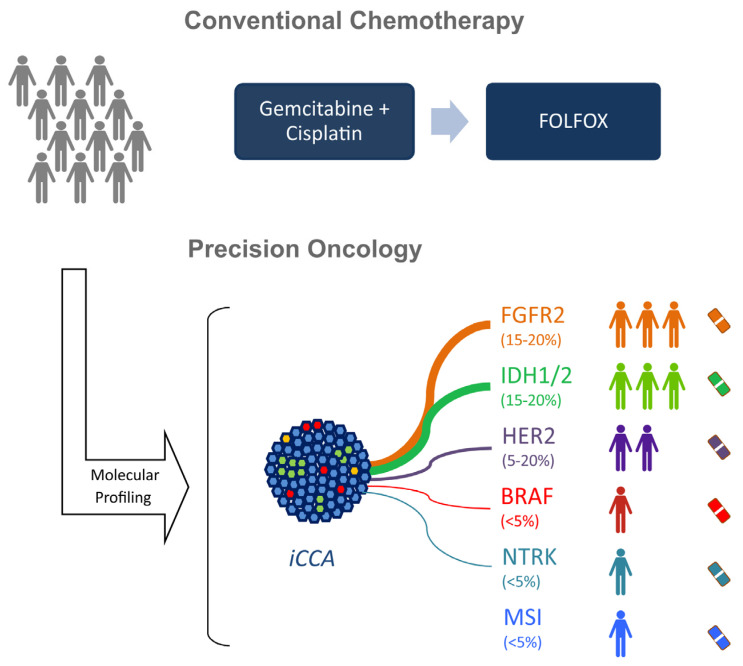
Conventional and precision oncological treatment approaches in advanced intrahepatic cholangiocarcinomas (iCCAs). proto-oncogene B-Raf (BRAF); human epidermal growth factor receptor 2 (Her2); Fibroblast Growth Factor Receptor gene 2 (FGFR 2), Isocitrate dehydrogenase 1 and 2 (IDH1/2); Neurotrophic tyrosine receptor kinase (NTRK); microsatellite instability (MSI).

Altogether, results of translational and early clinical studies outlined in Table 1 and Figure 2 clearly indicate that molecular testing and targeted therapies in selected patients might provide a major breakthrough for the outcome of CCA patients. Molecular approaches are currently being intensively pursued in multiple clinical trials. In the following section, we will discuss the current data on molecular therapeutic strategies for iCCA.

## 3. Molecular Targeted Therapies

### 3.1. FGFR2 Alterations

Fibroblast growth factor (FGF) signaling is involved in multiple cellular functions, including cell metabolism, development, as well as angiogenesis [38]. The fibroblast growth factor receptor (FGFR) family consists of four transmembrane receptors (FGFR1 to FGFR4), 18 FGF ligands, and a heparan sulfate proteoglycan that stabilizes and sequesters the FGFs. The ligand–receptor combination is responsible for the activation of various downstream signaling, such as RAS/RAF/MEK, JAK/STAT, and PI3K/AKT pathways [39]. Genetic alterations such as activating mutations, amplifications, or chromosomal translocations/fusions in the FGFR pathway contribute to malignant transformation. *FGFR2* fusions are the most frequent alterations detected in iCCA [22,40]. FGFR2 is located on chromosome 10 and, around 50% of *FGFR2* fusions evolve through intrachromosomal events. *FGFR2* fusions frequently result from chromosomal events that lead to an in-frame fusion between the 5′ end of the *FGFR2* gene and a partner gene, including the most prevalent *BICC1*, *PPHLN1*, *TACC3*, and *MGEA5*, leading to constitutive activation of the pathways [16,23].

As FGFR alterations represent potentially druggable targets for patients with iCCA, several studies have investigated the efficacy and safety of FGFR inhibitors in patients with iCCA and FGFR alterations, which are summarized in Table 1. Most importantly, a multicenter, open-label, single-arm, multicohort phase II study (FIGHT-202) tested the selective FGFR1-3 inhibitor pemigatinib in previously treated patients with iCCA [41]. The study enrolled 146 patients within three patient cohorts: 107 patients had *FGFR2* fusions or rearrangements (cohort A), 20 patients had other *FGF/FGFR* alterations (cohort B), and 18 showed no FGF/FGFR alterations (cohort C). Interestingly, none of the patients in cohorts B and C achieved a mentionable response to the treatment. In contrast, the ORR was 35.5% for patients with *FGFR2* fusions/rearrangements, and three patients achieved a complete response. Accordingly, patients of this subgroup had a promising mPFS of 6.9 months and an mOS of 21.2 months [41]. Toxicity profiles were overall manageable, with hyperphosphatemia occurring in 60% of the patients. The most common grade 3 or worse adverse events were hypophosphatemia (12%), arthralgia (6%), and stomatitis (5%). Overall, 45% had serious adverse events, which were most frequently abdominal pain (5%), pyrexia (5%), cholangitis (5%), and pleural effusion (3%). Based on these positive results, pemigatinib was recently approved by the United States Food and Drug Administration (FDA) and by the European Medicines Agency (EMA) for the second-line treatment of patients with advanced iCCA and *FGFR2* fusion/rearrangements.

Another early phase II trial investigated the antitumor activity of the alternative FGFR inhibitor infigratinib in previously treated patients with iCCA containing *FGFR2* fusions. Results of this study confirmed the encouraging efficacy observed within the FIGHT-202 study, with a high ORR of 30% and a prolonged mPFS and mOS of 6.8 and 12.5 months, respectively (Table 1) [24]. The toxicity profile of infigratinib was similar to that of pemigatinib, with hyperphosphatemia being the most common AE. High ORR (30%) and DCR (>80%) rates could be further demonstrated in three other early clinical trials that investigated pan-FGFR inhibitors in smaller Western and Asian patient cohorts [25,26,27]. Based on these positive findings, three randomized controlled phase III trials are currently recruiting patients with FGFR2 fusions/rearrangements that compare gemcitabine and cisplatin with infigratinib (PROOF) [42], pemigatinib (Fight-302) [43], or futibatinib (FOENIX-CCA3) [44] in the first-line setting. The designated primary endpoint is a prolongation of PFS.

Important upcoming challenges in the clinical care of patients will involve treatment resistance. Activity of most FGFR inhibitors depends on binding to the ATP-binding pocket of the tyrosine kinase. Gate keeper mutations can prevent the drug from accessing the hydrophobic pocket due to steric hindrance, and off-target resistance can impair long-lasting response to FGFR inhibitors by acquiring new (epi-) genomic alterations that lead to activation of alternative signaling pathways [45]. In addition, the co-mutational status of the tumors is another relevant factor that may influence treatment response and has to be considered [46].

### 3.2. IDH Mutations

In addition to *FGFR2* fusions, mutations of isocitrate dehydrogenase 1 and 2 (IDH1 and IDH2) represent clinically significant alterations in a sizeable number of iCCAs. Interestingly, *FGR2* translocation has been reported as mutually exclusive with *IDH1* mutations [19].

IDH1 and IDH2 encode for metabolic enzymes, and mutations lead to accumulation of the oncometabolite 2-hydroxyglutarate (2-HG), particularly inducing epigenetic changes in the tumors, such as DNA hypermethylation and altered expression of chromatin remodelers [47,48]. Comparing the prognosis of the molecular subtypes suggested a favorable prognosis for patients with *IDH* mutations in comparison to fluke-related iCCA [18] and in comparison to patients with *KRAS* and *TP53* mutations [12,49].

Currently, several IDH inhibitors have been investigated in clinical trials, such as ivosidenib (*IDH1*) and enasidenib (*IDH2*). A phase I dose escalation and expansion study tested ivosidenib in 73 patients with *IDH1* mutations that had progressed on one or more previous lines of therapy [28,29]. During the dose escalation phase, no dose-limiting toxicities were reported. The most common adverse events were fatigue (43%), nausea (25%), diarrhea (32%), and abdominal pain (20%). The median PFS reached 3.8 months (95% CI = 3.6–7.3) and mOS 13.8 months (95% CI = 11.1–29.3), which rendered IDH1 a promising therapeutic target in respective patients. Results of the subsequent phase III ClarIDHy study have been recently reported [50]. In this multicenter, randomized, double-blind, placebo-controlled trial, 185 patients with IDH1-mutated CCAs were randomly (2:1) assigned to ivosidenib (*n* = 124) or placebo (*n* = 61). The median PFS was significantly longer in the ivosidenib arm in comparison to placebo (2.7 vs. 1.4 months; HR = 0.37; *p* < 0.0001). The median OS reached 10.8 vs. 9.7 months, although crossover was permitted on progression (HR = 0.69; *p* = 0.060). The most common AEs were nausea (32.1%), diarrhea (28.8%), and fatigue (23.7%). Interestingly, the most common grade 3 or higher AEs were ascites (9.0%), blood bilirubin increase (5.4%), and anemia (7.2%) [29]. Overall, the data show a clear clinical benefit of IDH inhibition in previously treated patients with IDH mutant iCCA, confirming this pathway as another prime molecular target in iCCA.

### 3.3. BRAF Mutations

*BRAF* mutations in CCA involve mainly the *BRAF^V600E^* mutations that occur in the kinase domain at amino acid V600. Similar to the observations in a wide range of solid tumors, BRAF mutations activate the oncogenic MAPK signaling pathway and infer a particularly poor prognosis [50]. BRAF inhibitors such as dabrafenib and vemurafenib have been approved for *BRAF^V600E^*-mutated melanomas. Interestingly, the efficacy of monotherapy in other solid tumors such as metastatic colorectal cancer (mCRC) is limited [51,52,53]. Studies on resistance mechanisms suggest feedback activation of EGFR signaling [53,54], and therefore, combination treatments of BRAF inhibitors with MEK inhibitors have been tested and have resulted in positive results in solid tumors such as mCRC [55]. In iCCA, mutations in *BRAF* are rare (1–5%). A current phase II open-label, single-arm, multicenter, Rare Oncology Agnostic Research (ROAR) basket trial for patients with *BRAF^V600E^*-mutated rare cancers included 43 patients with BTC regardless of the anatomical location of CCA [32]. The results demonstrated that the combination of BRAF inhibition by dabrafenib plus MEK inhibition by trametinib resulted in high independent-reviewer-assessed ORRs of 47% (95% CI = 31–62). The most common grade 3–4 AE was increased γ-glutamyltransferase in five (12%) patients. The study suggests a promising activity of combination treatment with a manageable safety profile for patients with *BRAF^V600E^*-mutated CCAs.

### 3.4. HER2 Alterations

While the human epidermal growth factor receptor (HER2) has been well established as a predictive marker for HER2-targeted therapies in breast and gastric cancer, data on HER2 inhibition in BTC is limited [56]. Overall, alterations (overexpression/mutations/amplifications) of HER2 in BTC occur mainly in eCCA (19%) compared to iCCA (<5%) [56], and data of clinical trials exist only for BTC regardless of CCA subtypes. An early-phase basket trial with a small number of patients reported activity of dual inhibition with trastuzumab and pertuzumab in *HER2*-overexpressed or *HER2*-mutated BTC. Four of eleven patients had a partial response to dual treatment [30,31]. Ongoing is a phase II trial that is investigating the efficacy and safety of trastuzumab in combination with gemcitabine plus cisplatin (NCT03613168). Furthermore, an antibody–drug conjugate of an anti-HER2 antibody and a topoisomerase I inhibitor (trastuzumab deruxtecan (DS-8201)) is being evaluated in a phase II trial on BTC [57]. The data of the studies will further clarify the role of HER2 inhibition in BTC.

### 3.5. NTRK Fusions

Neurotrophic tyrosine receptor kinase (NTRK) gene fusions have been identified as oncogenic drivers in several adult and pediatric tumor entities. Larotrectinib and entrectinib gained approval for solid tumors with *NTRK* gene fusion regardless of the tumor entity based on the high response rates of up to 75% and durable disease control [33] with 1-year PFS rates of 55% [34]. Although rgw incidence of NTRK fusion genes is low (<5%) in iCCA, the high efficacy of specific inhibition warrants molecular testing in treatment refractory patients.

## 4. Immunotherapy

The increasing clinical implementation of immunotherapy (IT) among patients with various cancer types has led to intensive investigation of IT in patients with CCA. Up to now, results indicate that mono-IT provides only modest efficacy in unselected patients with CCA. As one of the first, the phase II, single-arm, multicohort KEYNOTE-158 study tested the programmed cell death protein-1 (PD-1) inhibitor pembrolizumab in previously treated patients with advanced solid tumors [15]. The subgroup of patients with BTC (*n* = 104) had an mPFS and mOS of 2.0 and 7.4 months, respectively, which is comparable to the results obtained by chemotherapy in the second-line setting. Regarding molecular biomarkers, programmed cell death protein-1 ligand 1 (PD-L1) expression was not a strong predictor of tumor response in this investigation. Consistently, the ORR was similar for PD-L1 expressers (*n* = 61) and PD-L1 non-expressers (*n* = 34): 6.6% (4/61) and 2.9% (1/34) respectively. In contrast, patients whose tumor harbored mismatch repair deficiency (dMMR)/microsatellite instability (MSI) had a clear benefit of IT, with an ORR of 40.9%, an mPFS of 4.2 months, and an mOS of 24.3 months, similarly as seen in other tumor entities [14]. However, restricting the predictive value of dMMR/MSI in CCA, the incidence of dMMR/MSI is generally low, i.e., less than 2%.

A competing, conceptionally similar phase II study investigated the PD-1 inhibitor nivolumab in 54 pretreated patients with CCA [13]. The ORR was 22%, the mPFS was 3.7 months, and the mOS was 14.2 months. In contrast to the KEYNOTE-158 study, here, tumoral PD-L1 expression was highly associated with a prolonged PFS (HR = 0.23; 95% CI = 0.10–0.51; *p* < 0.001). Interestingly, all patients who responded to treatment had mismatch repair protein-proficient tumors. Overall, results of the monotherapeutic approaches did not reveal a clear signal for response of CCA patients without dMMR/MSI status. These results highlight the need for molecular subclassification as well as combination therapies. Consistently, both IT combinations as well as IT and tyrosine kinase inhibitor (TKI) combinations are currently being pursued for patients with CCA in clinical trials. The phase II MEDITREME study was a biomarker phase II study that investigated the combination of gemcitabine and cisplatin with the PD-L1 antibody durvalumab with and without the cytotoxic T-lymphocyte-associated protein-4 (CTLA-4) antibody tremelimumab in 121 patients with BTC [35]. Encouragingly, none of the patients progressed under this therapy in the arm receiving durvalumab and gemcitabine plus cisplatin (100% disease control rate) and 73.4% had an objective tumor response. These impressive response rates were also transferred into a prolongation of survival with an mPFS of 11 months and an mOS of 18.1 months. Interestingly, the addition of tremelimumab in this trial seemed not to provide any additional benefit. Increased PD-L1 expression in tumor samples obtained after the first cycle of gemcitabine and cisplatin correlated with an improved mPFS (not achieved vs. 9.5 months; HR = 0.22; 95% CI = 0.03-1.71; *p* = 0.11). In contrast, the tumor mutational burden (TMB) did not correlate with the PFS or OS. Based on these promising results, combinations of IT with chemotherapy are further being investigated within the phase III TOPAZ-1 (NCT03875235) and KEYNOTE-966 (NCT04003636) trials, in which patients are randomized to gemcitabine and cisplatin alone or in combination with durvalumab or pembrolizumab, respectively.

The combination of IT with TKI is another strategy that is currently gaining increasing interest. The phase II LEAP-005 trial investigated the combination of the TKI lenvatinib with pembrolizumab in previously treated patients with advanced solid tumors [36]. For patients with BTC, the ORR was 10% and the mPFS was 6.1 months, providing promising activity. Moreover, the addition of chemotherapy to the combination of IT and TKI appears to achieve stronger antitumoral effects. In a recent phase II study, patients with advanced iCCA were treated with Lenvatinib, in addition to the PD1 inhibitor toripalimab, and gemcitabine and oxaliplatin [37]. Astonishingly, the disease control rate was 93.3%, the ORR was 80%, and both mPFS and mOS were not reached in the interim analysis. In concordance with previous results, the ORR was significantly associated with PD-L1 expression (100% vs. 68.8%; *p* = 0.048) and DNA damage repair-related mutations (95.0% vs. 55.6%; *p* = 0.002) in tumor samples, again underlining the potential benefit of enrichment of patients based on their molecular profile to increase the efficacy of the treatment.

In summary, mono-IT provides modest efficacy in unselected patients with iCCA. Combinations of IT with TKI and/or chemotherapy in early clinical investigations demonstrate promising results. As of today, no clear biomarker of molecular profiles could be identified to predict the response to IT and IT combinations. However, despite the low frequency, MSI might be a good predictor of response. PD-L1 expression in tumors may also predict a better response, but results of various studies are not fully conclusive. Current data therefore cannot support patient selection based on tumoral PD-L1 expression. Results of larger biomarker trials are urgently being awaited, and further implementation of translational studies, e.g., subtyping and enrichment, seems highly promising.

## 5. Conclusions

The prognosis of patients with BTC remains dismal. Large phase III studies over the past 10 years have established chemotherapy as a standard of care in adjuvant and palliative disease stages in, with respect to anatomy and molecular alterations, unselected patients with CCA [4,7,8,9,11]. Results from molecular profiling studies demonstrate that BTCs are a group of molecularly highly distinct tumor types with different relevant subclasses. Frequent identification of druggable oncogenic alterations and tumors with dominant activation of immune response signaling provide a clear rationale for personalized treatment approaches, in addition to classical chemotherapies [17,18,19,20,21]. Various aberrations have been characterized. Especially in iCCAs, specific subtypes have been associated with oncogenic driver alterations such as *FGFR2* fusions/rearrangement and *IDH1* mutations, which renders iCCA a paradigm for precision oncological approaches [17,18,19,20,21]. The efficacy and safety of multiple inhibitors have now been investigated in clinical trials, and molecular stratified treatments have reached evidence of phases II and III [24,27,29,32,41,57]. Pemigatinib is the first FGFR2 inhibitor that has been approved by the FDA and the EMA and represents the first second-line chemotherapy-free treatment for patients with *FGFR2* fusions or rearrangements [41]. The promising results of phase II studies on FGFR2 inhibitors have led to subsequent phase III studies that now evaluate the efficacy of FGFR2 inhibitors against the standard of care in first-line settings. Recent evidence also clearly supports the use of the IDH1 inhibitor ivosidenib in IDH1-mutated patients. Other oncogenic targets include *BRAF*, *NTRK*, and *HER2*. In addition, first signals from IT-based approaches indicate the potential benefit of these approaches in selected patients. Although IT–IT and IT–TKI combinations demonstrated promising antitumor activity with a manageable safety profile in treatment-experienced patients [29], no predictive biomarkers currently exist.

Recent comprehensive integrative genomic analyses have grouped iCCAs into relevant subclasses. In the context of precision oncology, two molecular classes are relevant [58]—the proliferation class with activation of oncogenic driver alterations, including mutations in *KRAS* and *RAF* and association with a worse outcome, as well as an inflammation class with activation of inflammatory signaling pathways, overexpression of cytokines, and STAT3 activation. To identify effective biomarkers and promote molecular-driven treatment approaches for iCCA patients, implementation of rigorous molecular testing and enrichment of patients as well as a consistent stratification of patients in clinical trials seem promising and will likely improve patient outcomes.

## Figures and Tables

**Table 1:** Clinical trials based on molecular alterations or immuno-oncological approaches for BTC.

Study	Phase	Therapy	Molecular Alteration	*n*	ORR % (95% CI)	DCR % (95% CI)	mPFS Months (95% CI)	mOS Months (95% CI)	Ref.
Fight-202	2	Pemigatinib (FGFR 1–3)	Cohort A: FGFR2 fusions/rearrangements Cohort B: Other FGFR2 alterations Cohort C: No FGFR2 alterations	107 *	35.5 (26.5–45.4)	82 (74–89)	6.9 (6.2–9.6)	21.1 (14.8–NE)	[22,23]
-	2	Infigratinib (FGFR 1–3)	FGFR2 fusions	71	31 (20.5–43.1)	83.6 (72.5–91.5)	6.8 (5.3–7.6)	12.5 (9.9–16.6)	[24]
	2	Erdafitinib (pan-FGFR)	FGFR2 aberrations/fusions	11	66.7 (N/A)	100 (N/A)	5.59 (1.87–NE)	N/A	[25]
-	2	Derazantinib (pan-FGFR)	FGFR2 fusions	29	20.7 (N/A)	82.8 (N/A)	5.7 (4.0–9.2)	NR	[26]
FOENIX-CCA2	2	Futibatinib (pan-FGFR)	FGFR2 fusions/rearrangements	67	34.3 (N/A)	76.1 (N/A)	N/A	N/A	[27]
-	2	Ivosedinib	IDH1 mutations	73	5 (1.5–13.4)	61 (N/A)	3.8 (3.6–7.3)	13.8 (11.1–29.3)	[28]
ClarIDHy	3	Ivosedinib vs. placebo	IDH1 mutations	185	2.4 (N/A)	53 (N/A)	2.7 vs. 1.4 (1.6–4.2)	10.8 vs. 6.0 (7.7–17.6)	[29]
-	2	Pertuzumab + trastuzumab	Her2 amplification/overexpression	11	36.4 (N/A)	63.6 (N/A)	4.2 (1.2–5.4)	N/A	[30,31]
ROAR	2	Dabrafenib + trametinib	BRAF^V600E^ mutations	43	47 (31–62)	N/A	7.2 (N/A)	11.2 (N/A)	[32]
-	1–2	Larotrectinib	TRK fusion	55 (2 CCA)	75 (61–85)	88 (N/A)	NR	NR	[33,34]
KEYNOE-158	2	Pembrolizumab		104	5.8 (2.1–12.1)	22.1 (N/A)	2 (1.9–2.1)	7.4 (5.5–9.6)	[15]
	2	Nivolumab		54	11 (N/A)	50 (N/A)	3.7 (2.30–5.69)	14.2 (5.98–NR)	[13]
Meditreme	2	Cohort A:Gem + Cis + D + T Cohort B:Gem + Cis + D + T Cohort C:Gem + Cis + D		121	A: 50 (32.1–67.9) B: 73.4 (60.5–86.3) C: 73.3 (60.4–86.2)	A: 96.7 (90.3–100) B: 100 (100–100) C: 97.8 (93.5–100)	A: 13.0 (10.1–15.9) B: 11 (7.0–15.0) C: 11.9 (10.1–13.7)	A: 15 (10.7–19.3) B: 18.1 (11.3–24.9) C: 20.7 (13.8–27.6)	[35]
LEAP-005	2	Lenvatinib + pembrolizumab	-	31	10 (2–26)	68 (N/A)	6.1 (2.1–6.4)	N/A	[36]
	2	Lenvatnib + toripalimab + Gem + Cis		30	80 (61.4–92.3)	93.3 (77.9–99.2)	NR	NR	[37]

* Cohort A; Cis, cisplatin; D, durvalumab; DCR, disease control rate; FGFR, fibroblast growth factor receptor; Gem, gemcitabine; Her2, human epidermal growth factor receptor 2; IDH, isocitrate dehydrogenase; mOS, median overall survival; mPFS, median progression-free survival; NR, not reached; NE, not assessed; ORR, overall response rate; T, tremelimumab; TRK, neurotrophic tyrosine receptor kinases.

## Data Availability

No new data were created or analyzed in this study. Data sharing is not applicable to this article.

## Abbreviations

ASCO	American Society of Clinical Oncology
BSC	best supportive care
BTC	biliary tract cancer
CCA	cholangiocarcinoma
CI	confidence interval
CTLA-4	cytotoxic T-lymphocyte-associated protein-4
eCCA	extrahepatic cholangiocarcinoma
FDA	United States Food and Drug Administration
FGFR	fibroblast growth factor receptor
GBCA	gallbladder carcinoma
dMMR	mismatch repair deficiency
iCCA	intrahepatic cholangiocarcinoma
IDH	isocitrate dehydrogenase
FU	fluoropyrimidine
HR	hazard ratio
IT	immunotherapy
ITT	intention to treat
MMR	mismatch repair
mOS	median overall survival
MSI	microsatellite instability
mRFS	median recurrence-free survival
ORR	overall response rate
OS	overall survival
PD-1	programmed cell death protein-1
PD-L1	programmed cell death protein-1 ligand 1
QOL	quality of life
RFS	recurrence-free survival
TKI	tyrosine kinase inhibitor
vs.	versus
